# The delta neutrophil index predicts development of multiple organ dysfunction syndrome and 30-day mortality in trauma patients admitted to an intensive care unit: a retrospective analysis

**DOI:** 10.1038/s41598-018-35796-4

**Published:** 2018-11-30

**Authors:** Taeyoung Kong, Yoo Seok Park, Hye Sun Lee, Sinae Kim, Jong Wook Lee, Je Sung You, Hyun Soo Chung, Incheol Park, Sung Phil Chung

**Affiliations:** 1 Department of Emergency Medicine, Yonsei University College of Medicine, Seoul, Republic of Korea; 2 Department of Emergency Medicine, Graduate School of Medicine, Kangwon National University, Chuncheon, Republic of Korea; 3 Department of Research Affairs, Biostatistics Collaboration Unit, Yonsei University College of Medicine, Seoul, Republic of Korea; 4 Department of Laboratory Medicine, Konyang University Hospital, Daejeon, Republic of Korea

## Abstract

No studies have examined the role of delta neutrophil index (DNI) reflecting on immature granulocytes in determining the severity of multiple organ dysfunction (MODS) and short-term mortality. This study investigated the utility of the automatically calculated DNI as a prognostic marker of severity in trauma patients who were admitted to an intensive care unit (ICU). We retrospectively analysed prospective data of eligible patients. We investigated 366 patients. On multivariable logistic regression analysis, higher DNI values at 12 h (odds ratio [OR], 1.079; 95% confidence interval [CI]: 1.037–1.123; *p* < 0.001) and 24 h were strong independent predictors of MODS development. Multivariable Cox regression analysis revealed that increased DNI at 12 h (hazard ratio [HR], 1.051; 95% CI, 1.024–1.079; *p* < 0.001) was a strong independent predictor of short-term mortality. The increased predictability of MODS after trauma was closely associated with a DNI > 3.25% at 12 h (OR, 12.7; 95% CI: 6.12–26.35; *p* < 0.001). A cut-off of >5.3% at 12 h was significantly associated with an increased risk of 30-day mortality (HR, 18.111; 95% CI, 6.988–46.935; *p* < 0.001). The DNI is suitable for rapid and simple estimation of the severity of traumatic injury using an automated haematologic analyser without additional cost or time.

## Introduction

Despite recent therapeutic advances, the World Health Organization assesses that injury causes 5.8 million death annually, and that this number is increasing worldwide^1,2^. Trauma-related mortality and disability is a major public health issue^2,3^. Moreover, the distribution of the timing of traumatic deaths is tri-modal^3^: traumas owing to non-salvageable injuries result in immediate deaths^4^; haemorrhagic or neurologic conditions, including haemorrhagic injuries of abdominal organs or expanding intracranial mass lesions, cause early death within the first 24 h following severe trauma^4,5^; and complications such as disseminated intravascular coagulopathy, sepsis, acute respiratory distress syndrome, and multiple organ dysfunction syndrome (MODS) cause late deaths days or weeks after injury in many trauma victims^4,5^. A strong immune response to traumatic injury is considered a critical factor in eliciting life-threatening conditions^5^. MODS remains the primary cause of late death after severe trauma, including up to 30% of deaths that are deemed possibly preventable^6,7^. Patents with MODS experience increased lengths of stay in intensive care units (ICUs) and may require renal replacement therapy^6,7^. Therefore, clinical improvement and survival in patients with major traumatic injuries require early recognition of their conditions and interventions to prevent the development of MODS^6,7^.

Technological advances in automated haematological analysers have enabled the acquisition of the delta neutrophil index (DNI) using leukocyte differentials obtained from two independent channels, the myeloperoxidase tungsten-halogen channel and the lobularity/nuclear density channel^8,9^. The DNI reflects the proportion of circulating immature granulocytes and is calculated as the difference between the leukocyte differentials measured in the myeloperoxidase channel and the differentials detected in the lobularity channel^8,9^. A previous study showed that the DNI (as calculated using an automated blood cell analyser) was strongly correlated with the manual immature granulocyte counts (r = 0.75, *p* < 0.05)^10^; an increase in the number of immature granulocytes in circulation is an important criterion in the diagnosis of systemic inflammatory response syndrome^11^. Several studies have found that a higher DNI value is closely associated with a positive blood culture, septic shock, disseminated intravascular coagulation, and mortality in critical care patients with suspected sepsis^10–13^. Recently, an increased DNI was also shown to reflect the severity of sterile inflammation-linked diseases such as out-of-hospital cardiac arrest, upper gastrointestinal haemorrhage, pulmonary embolism, and acute myocardial infarction^8,14,15^.

In patients with traumatic injury, systemic inflammatory response syndrome is abruptly initiated within 30 minutes of major traumatic injury, and is an inflammatory response to hypoperfusion and reperfusion owing to blood loss and tissue damage rather than infection^2^. As a result, the systemic response to severe injury leads to multiorgan failure (MOF) and sepsis that compounds the original traumatic injury^2^. Tissue injury after trauma releases damage-associated molecular patterns from necrotic cells, which activate immune cells such as neutrophils^2^. Increased pro-inflammatory cytokines activate neutrophils that elicit the destruction of healthy organ tissues with proteases and reactive oxygen species, and also sequestrate neutrophils in bystander organs by migrating across damaged endothelium^2^. Major loss of blood components, including neutrophils, occurs following an injury owing to vascular damage and massive haemorrhage^16,17^. To compensate for the tremendous depletion and demand for activated neutrophils following their marked loss owing to consumption, sequestration, and destruction of mature cells, the switch to emergency granulopoiesis considerably enhances the release of immature granulocytes by rapid production and turnover of cells into the peripheral blood from the bone marrow^16–19^. As a consequence of severe inflammation, the dysregulated neutrophil function, known as neutrophil paralysis, further impairs the migration of neutrophils, and causes unwanted antimicrobial responses and neutrophil sequestration in remote organs^20^. In patients with severe traumatic injuries such as haemorrhage and damage to organs and tissues, the release of immature granulocytes into circulation may contribute to the failure to protect against infection and the risk of MOF^21,22^.

To the best of our knowledge, no study has assessed the ability of serial DNI measurements to predict the clinical severities of MODS and short-term mortality, nor have any DNI cut-off values that predict the severity of trauma patients admitted to an ICU been derived. Hence, in this study, we investigated the clinical utility of the DNI as a predictor of severity in trauma patients admitted to an ICU.

## Results

### Study population and clinical evaluation

**Figure 1:**
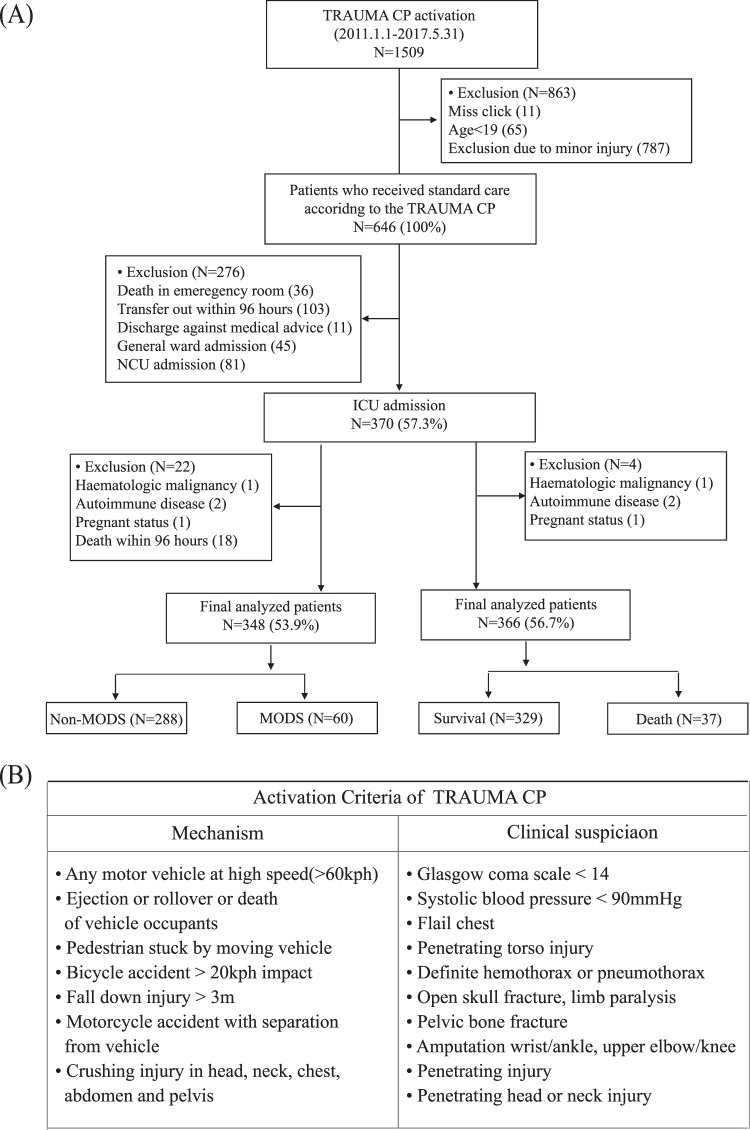
Flow diagram of patient enrolment (**A**) and the criteria for trauma critical pathway activation (**B**).

**Figure 2:**
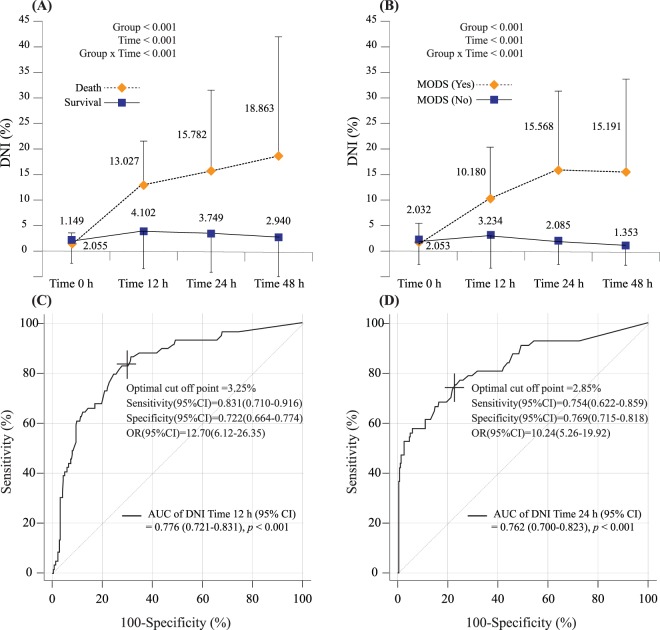
Linear mixed model of the delta neutrophil index (DNI) to estimate significant differences between groups over time according to 30-day mortality (**A**) and development of multiple organ dysfunction syndrome (MODS) (**B**). The receiver operating characteristic curves for predictability of the DNI at 12 h (**C**) and 24 h (**D**) after admission according to MODS development.

Figure 1 shows the enrolment and clinical outcomes of trauma patients admitted to the ICU. 366 patients (mean age 50.5 ± 18.8 years) with a mean ISS of 28.7 ± 11.5 were enrolled. Thirty-seven patients (10.1%) had died by day 30. The ISS was significantly greater in the non-survival group (41.5 ± 13.8) than in the survival group (27.3 ± 10.3; *p* < 0.001). Sixty patients (17.2%) developed MODS in the ICU (Table 1). The linear mixed model suggested that changes in DNI values from ED admission to 48 h later were different between the groups over time; there were significant changes in DNI values as correlated with 30-day mortality and MODS (Fig. 2A,B).

**Table 1:** Clinical characteristics of patients stratified by 30-day mortality and development of multiple organ dysfunction syndrome.

Variables	30-day Mortality				Development of MODS			
	Total N = 366 (100%)	Survival N = 329 (89.9%)	Death N = 37 (10.1%)	*P*	Total N = 348 (100%)	No N = 288 (82.8%)	Yes N = 60 (17.2%)	*P*
Age (years)	50.536 ± 18.758	49.687 ± 18.548	58.081 ± 19.172	0.01*	50.046 ± 18.776	49.785 ± 18.685	51.300 ± 19.317	0.57
Male sex [n (%)]	264(72.13)	238(72.34)	26(70.27)	0.79	251(72.13)	204(70.83)	47(78.33)	0.239
BMI (kg/m^2^)	23.883 ± 3.532	23.833 ± 3.529	24.331 ± 3.573	0.417	23.893 ± 3.564	23.838 ± 3.529	24.159 ± 3.746	0.529
ISS (point)	28.697 ± 11.497	27.255 ± 10.275	41.514 ± 13.803	<0.001*	28.023 ± 11.062	26.201 ± 9.258	36.767 ± 14.452	<0.001*
APACHE II score (point)	11.59 ± 7.099	10.416 ± 5.943	22.027 ± 8.036	<0.001*	11.003 ± 6.621	9.809 ± 5.549	16.733 ± 8.227	<0.001*
SOFA score (point)	4.88 ± 3.947	4.161 ± 3.35	11.27 ± 3.006	<0.001*	4.486 ± 3.602	3.451 ± 2.749	9.450 ± 3.039	<0.001*
**Initial vital sign**								
Mean blood pressure (mmHg)	82.165 ± 24.224	84.966 ± 21.476	57.261 ± 32.224	<0.001*	83.727 ± 22.819	85.770 ± 21.557	73.922 ± 26.153	0.002*
Heart rate (bpm)	92.336 ± 24.365	93.267 ± 20.879	84.054 ± 44.369	0.22	92.894 ± 23.278	92.219 ± 21.691	96.133 ± 29.752	0.337
Respiratory rate (bpm)	17.915 ± 4.178	18.085 ± 3.819	16.405 ± 6.444	0.128	17.615 ± 3.8	18.101 ± 3.556	17.167 ± 5.823	0.236
Body temperature (°C)	36.346 ± 0.64	36.372 ± 0.65	36.119 ± 0.5	0.022*	36.361 ± 0.646	36.431 ± 0.590	36.027 ± 0.788	<0.001*
Mental change [n (%)]	67(18.31)	47(14.29)	20(54.05)	<0.001*	56(16.09)	31(10.76)	25(41.67)	<0.001*
**Mechanism [n (%)]**				0.125				0.749
Motor vehicle accident at high speed	45(12.3)	42(12.77)	3(8.11)		44(12.64)	34(11.81)	10(16.67)	
Ejection or rollover of vehicle	14(3.83)	13(3.95)	1(2.7)		13(3.74)	10(3.47)	3(5)	
Pedestrian stuck by moving vehicle	111(30.33)	94(28.57)	17(45.95)		101(29.02)	83(28.82)	18(30)	
Bicycle accident > 20 km impact	7(1.91)	5(1.52)	2(5.41)		7(2.01)	6(2.08)	1(1.67)	
Fall > 3 m	86(23.5)	76(23.1)	10(27.03)		80(22.99)	65(22.57)	15(25)	
Motorcycle accident	60(16.39)	58(17.63)	2(5.41)		60(17.24)	53(18.4)	7(11.67)	
Crushing injury	19(5.19)	18(5.47)	1(2.7)		19(5.46)	15(5.21)	4(6.67)	
Stab wound	24(6.56)	23(6.99)	1(2.7)		24(6.9)	22(7.64)	2(3.33)	
**Comorbidity [n (%)]**								
Hypertension	107(29.23)	94(28.57)	13(35.14)	0.405	91(26.15)	73(25.35)	18(30.00)	0.456
Diabetes mellitus	65(17.76)	56(17.02)	9(24.32)	0.27	53(15.23)	43(14.93)	10(16.67)	0.734
Chronic pulmonary disease	23(6.28)	21(6.38)	2(5.41)	>0.999	21(6.03)	17(5.90)	4(6.67)	0.769
Cardiovascular disease	24(6.56)	21(6.38)	3(8.11)	0.723	23(6.61)	18(6.25)	5(8.33)	0.568
Old cerebrovascular accident	7(1.91)	7(2.13)	0(0.00)	>0.999	7(2.01)	6(2.08)	1(1.67)	0.999
Malignancy	9(2.46)	8(2.43)	1(2.70)	>0.999	8(2.30)	8(2.78)	0(0.00)	0.36
Chronic liver disease	5(1.37)	3(0.91)	2(5.41)	0.082	4(1.15)	2(0.69)	2(3.33)	0.139
Chronic kidney disease	7(1.91)	7(2.13)	0(0.00)	>0.999	7(2.01)	6(2.08)	1(1.67)	0.999
**Treatment within 24 hours [n (%)]**								
Surgery	135(36.89)	112(34.04)	23(62.16)	<0.001*	122(35.06)	92(31.94)	30(50.00)	0.008*
Embolization	71(19.40)	59(17.93)	12(32.43)	0.035*	64(18.39)	47(16.32)	17(28.33)	0.029*
Conservative management	179(48.91)	170(51.67)	9(24.32)	0.002*	177(50.86)	156(54.17)	21(35.00)	0.007*
**Transfusion in first 24 hours**								
Packed red blood cells (Unit)	4.156 ± 8.185	2.964 ± 6.525	14.757 ± 12.764	<0.001*	3.184 ± 6.584	1.917 ± 3.949	9.267 ± 11.561	<0.0001*
Fresh Frozen Plasma (Unit)	2.806 ± 6.561	1.875 ± 4.947	11.081 ± 11.625	<0.001*	2.043 ± 5.021	1.104 ± 3.356	6.550 ± 8.278	<0.0001*
Platelet concentrate (Unit)	2.593 ± 6.451	1.733 ± 5.327	10.243 ± 9.825	<0.001*	1.931 ± 5.445	0.819 ± 2.847	7.267 ± 9.998	<0.0001*
**Laboratory data**								
White blood cell count (10^3^/μL)	12.748 ± 5.073	12.884 ± 5.147	11.538 ± 4.235	0.126	12.864 ± 5.113	13.023 ± 5.164	12.104 ± 4.829	0.206
Hemoglobin (g/dL)	12.94 ± 2.344	13.056 ± 2.313	11.914 ± 2.4	0.005*	13.017 ± 2.34	13.069 ± 2.285	12.770 ± 2.598	0.369
Platelet count (10^3^/μL)	231.86 ± 77.021	234.27 ± 75.407	210.43 ± 88.387	0.074	233.22 ± 77.358	236.649 ± 74.141	216.750 ± 90.126	0.114
PT (INR)	1.033 ± 0.235	1.012 ± 0.186	1.215 ± 0.454	0.011*	1.021 ± 0.203	1.005 ± 0.184	1.098 ± 0.266	0.012*
BUN (mg/dL)	16.896 ± 6.707	16.712 ± 6.629	18.541 ± 7.262	0.116	16.818 ± 6.571	16.889 ± 6.730	16.475 ± 5.786	0.658
Creatinine (mg/dL)	0.995 ± 0.734	0.967 ± 0.726	1.244 ± 0.77	0.029*	0.979 ± 0.712	0.944 ± 0.699	1.151 ± 0.755	0.040*
AST (IU/L)	136.64 ± 180.69	130.84 ± 168.15	188.19 ± 265.28	0.206	132.99 ± 166.17	122.372 ± 155.957	183.933 ± 202.231	0.029*
ALT (IU/L)	92.975 ± 143.21	89.134 ± 128.83	127.14 ± 235.35	0.34	90.397 ± 127.58	83.396 ± 120.905	124.000 ± 152.402	0.056
Lactate (mmol/L)	3.941 ± 3	3.562 ± 2.58	7.178 ± 4.209	<0.001*	3.811 ± 2.905	3.333 ± 2.445	6.140 ± 3.757	<0.001*
Potassium (mmol/L)	4.045 ± 0.572	4.034 ± 0.542	4.141 ± 0.793	0.431	4.043 ± 0.557	4.050 ± 0.519	4.012 ± 0.713	0.697
tCO2 (mmol/L)	18.858 ± 3.534	19.119 ± 3.332	16.541 ± 4.394	0.001*	18.92 ± 3.479	19.299 ± 3.245	17.100 ± 3.986	<0.001*
DNI Time-0 (%)	1.963 ± 4.305	2.055 ± 4.483	1.149 ± 2.011	0.031*	2.049 ± 4.396	2.053 ± 4.547	2.032 ± 3.616	0.969
DNI Time-12 (%)	4.953 ± 8.379	4.102 ± 7.951	13.027 ± 8.138	<0.001*	4.469 ± 8.026	3.234 ± 6.833	10.180 ± 10.407	<0.001*
DNI Time-24 (%)	4.539 ± 9.319	3.749 ± 8.121	15.782 ± 16.114	0.002*	4.414 ± 9.279	2.085 ± 4.517	15.568 ± 15.927	<0.001*
DNI Time-48 (%)	3.888 ± 10.305	2.94 ± 8.013	18.863 ± 23.615	0.009*	3.877 ± 10.319	1.353 ± 3.877	15.191 ± 19.094	<0.001*

BMI, body mass index; ISS, injury severity score; APACHE II, Acute Physiology and Chronic Health Evaluation; SOFA, sequential organ failure assessment; PT, prothrombin time; BUN, blood urea nitrogen; AST, aspartate aminotransferase; ALT, alanine aminotransferase; DNI, delta neutrophil index. *P < 0.05.

### DNI as predictor of 30-day mortality and MODS development

Univariate Cox and logistic regression analyses revealed that DNI values were significantly different at 12 h in patients who died within 30 days or experienced MODS versus those who did not (Supplement 1). The multivariable Cox regression model demonstrated that an increased DNI value at 12 h after ED admission (HR, 1.051; 95% CI, 1.024–1.079; *p* < 0.001) was a strong independent predictor of short-term mortality among severe trauma patients admitted to an ICU (Table 2). On multivariable logistic regression analysis, higher DNI values at 12 and 24 h after ED admission were also strong independent predictors of the development of MODS (Table 3). The AUCs for predicting MODS using the DNI at 12 and 24 h among patients admitted to an ICU with severe trauma were 0.776 (*p*- < 0.001) and 0.762 (*p* < 0.001), respectively (Fig. 2C,D).

**Table 2:** Multivariable Cox proportional hazards regression analysis for predictors of 30-day mortality.

Variable	Multivariable cox proportional hazard regression analysis (30-day mortality)							
	HR (95% CI)	*P*	HR (95% CI)	*P*	HR (95% CI)	*P*	HR (95% CI)	*P*
ISS (per 1point)	1.026 (1.002–1.050)	0.032*	1.037 (1.012–1.062)	0.004*	1.024 (0.991–1.057)	0.154	1.031 (0.997–1.067)	0.075
APACHE II score (per 1point)	1.135 (1.072–1.201)	<0.001*	1.108 (1.042–1.178)	0.001*	1.128 (1.048–1.215)	0.001*	1.090 (1.004–1.184)	0.039*
Transfusion of pRBC (per 1unit)	1.012 (0.986–1.039)	0.375	1.019 (0.991–1.048)	0.181	0.990 (0.948–1.033)	0.629	0.978 (0.922–1.036)	0.446
**Treatment within 24 hours**								
*Surgery*	6.491 (1.611–26.158)	0.009*	4.009 (0.931–17.267)	0.062	5.150 (0.709–37.397)	0.105	4.556 (0.373–55.669)	0.235
*Embolization*	1.963 (0.777–4.958)	0.154	2.078 (0.792–5.450)	0.137	2.172 (0.523–9.026)	0.286	1.441 (0.257–8.092)	0.678
*Conservative management*	2.105 (0.444–9.982)	0.349	2.389 (0.444–12.865)	0.311	2.203 (0.265–18.314)	0.465	2.966 (0.210–41.858)	0.421
Prothrombin time (per 1 INR)	1.302 (0.528–3.206)	0.567	1.569 (0.575–4.281)	0.380	1.449 (0.364–5.763)	0.598	1.368 (0.252–7.414)	0.717
Lactate (per 1 mmol/L)	1.005 (0.903–1.118)	0.931	1.020 (0.910–1.144)	0.731	0.999 (0.872–1.145)	0.992	1.052 (0.907–1.219)	0.506
DNI Time-0 (per 1%)	0.867 (0.730–1.028)	0.101						
DNI Time-12 (per 1%)			1.051 (1.024–1.079)	<0.001*				
DNI Time-24 (per 1%)					1.020 (0.987–1.055)	0.231		
DNI Time-48 (per 1%)							1.043 (1.014–1.072)	0.003*

ISS, injury severity score; APACHE II, Acute Physiology and Chronic Health Evaluation; pRBC, packed red blood cells; DNI, delta neutrophil index; *P < 0.05.

**Table 3:** Multivariable logistic regression analysis for predictors of the development of multiple organ dysfunction syndrome.

Variable	Multivariable logistic regression analysis (Development of MODS)							
	OR (95% CI)	*P*	OR (95% CI)	*P*	OR (95% CI)	*P*	OR (95% CI)	*P*
ISS (per 1point)	1.047 (1.012–1.082)	0.008*	1.061 (1.023–1.101)	0.002*	1.045 (1.005–1.085)	0.026*	1.044 (1.006–1.084)	0.023*
APACHE II score (per 1point)	1.082 (1.017–1.151)	0.012*	1.065 (0.999–1.136)	0.055	1.058 (0.989–1.132)	0.101	1.062 (0.994–1.136)	0.075
Transfusion of pRBC (per 1unit)	1.102 (1.026–1.183)	0.008*	1.109 (1.029–1.196)	0.007*	1.089 (1.005–1.180)	0.038*	1.088 (1.009–1.174)	0.029*
**Treatment within 24 hours**								
*Surgery*	4.423 (0.919–21.280)	0.064	2.215 (0.421–11.650)	0.348	2.346 (0.349–15.775)	0.381	2.476 (0.361–16.992)	0.356
*Embolization*	3.472 (0.835–14.435)	0.087	2.920 (0.650–13.116)	0.162	2.384 (0.414–13.739)	0.331	2.536 (0.424–15.163)	0.308
*Conservative management*	3.644 (0.612–21.700)	0.156	3.847 (0.595–24.861)	0.157	3.545 (0.423–29.711)	0.243	3.109 (0.365–26.500)	0.299
AST (per 1 IU/L)	1.001 (0.999–1.003)	0.482	1.001 (0.999–1.003)	0.462	1.001 (0.998–1.003)	0.622	1.000 (0.998–1.003)	0.70
Prothrombin time (per 1 INR)	1.189 (0.290–4.880)	0.810	1.424 (0.328–6.189)	0.637	0.918 (0.174–4.847)	0.920	1.457 (0.304–6.977)	0.638
Lactate (per 1 mmol/L)	1.091 (0.959–1.242)	0.187	1.085 (0.947–1.242)	0.240	1.072 (0.930–1.235)	0.338	1.068 (0.927–1.231)	0.364
DNI Time-0 (per 1%)	0.996 (0.914–1.086)	0.934						
DNI Time-12 (per 1%)			1.079 (1.037–1.123)	<0.001*				
DNI Time-24 (per 1%)					1.125 (1.070–1.183)	<0.001*		
DNI Time-48 (per 1%)							1.121 (1.054–1.194)	<0.001*

ISS, injury severity score; APACHE II, Acute Physiology and Chronic Health Evaluation; pRBC, packed red blood cells; AST, aspartate aminotransferase; DNI, delta neutrophil index; *P < 0.05.

### Comparison of DNI and conventional clinical markers as predictors of 30-day mortality and MODS in critically ill trauma patients

**Figure 3:**
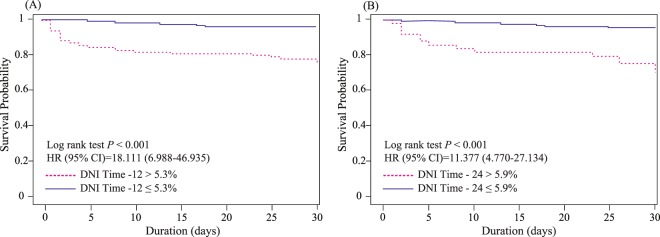
The delta neutrophil index (DNI) as a predictor of 30-day mortality. Higher DNI values at 12 h (**A**) and 24 h (**B**) after admission were significantly associated with an increased risk of 30-day mortality among trauma patients admitted to an ICU.

**Figure 4:**
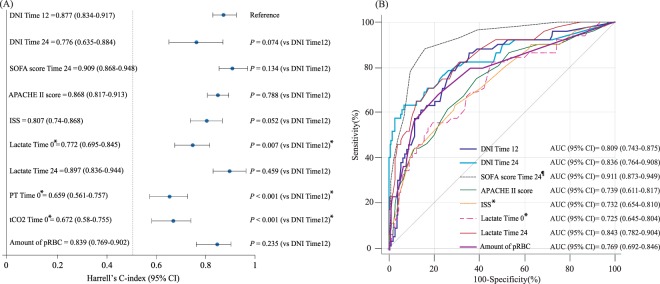
Comparison of Harrell’s C-index for the delta neutrophil index (DNI) for the prediction of 30-day mortality according to time (**A**); and comparison of the area under the curve (AUC) for the DNI when predicting the development of multiple organ dysfunction syndrome (MODS) according to time (**B**). The Harrell’s C-index and AUC showed discriminative abilities for the risk stratification of 30-day mortality and development of MODS (statistical information is shown in supplements 2 and 3). (**A**) *Harrell’s C-index of DNI at 12 h was statistically superior to lactate, prothrombin time, and tCO_2_ on emergency department admission (*p* < 0.05). (**B**) *AUC of DNI at 12 h was statistically superior to lactate on emergency department admission and Injury Severity Score (*p* < 0.05). ^¶^AUC of DNI at 12 h was statistically inferior to the Sequential Organ Failure Assessment score at 24 h after emergency department admission (*p* < 0.05).

To predict MODS development, ROC comparisons showed that the AUC of the DNI at 12 h was not significantly inferior to those of other markers (APACHE II, ISS, total transfused packed red blood cell volumes, and lactate on ED admission, and 24 h) except for the SOFA. Moreover, the DNI value at 24 h was not significantly inferior to those of other markers, including SOFA, APACHE II, and lactate, at 24 h. However, it was significantly superior to lactate and ISS on ED admission (Supplement 2). Using Youden’s method, the optimal cut-offs for the DNI values at 12 and 24 h were found to be 3.25% (sensitivity: 83.1 [71–91.6]; specificity: 72.2 [66.4–77.4]) and 2.85% (sensitivity: 75.4 [62.2–85.9]; specificity: 76.9 [71.5–81.8]), respectively. A DNI value > 3.25% was closely associated with the probability of MODS after trauma at 12 h after ED admission (OR, 12.7; 95% CI: 6.12–26.35; *p* < 0.001), as was a DNI value > 2.85% at 24 h (OR, 10.24; 95% CI: 5.26–19.92; *p* < 0.001) (Fig. 2C,D). To estimate the optimal cut-off values based on time-to-event data, we created Kaplan-Meier curves for 30-day mortality according to DNI values (Fig. 3); a log-rank test at 12 h after ED admission showed that these DNI cut-off values were independent predictors of 30-day mortality (*p* < 0.001). Harrell’s C-index for the prediction of 30-day mortality using the DNI was 0.876 (95% CI 0.831–0.916, *p* < 0.001) at 12 h after ED admission (Fig. 4A). The optimal DNI cut-off value for predicting 30-day mortality, the identification of which was a primary aim of this study, was 5.3% at 12 h after ED admission (*p* < 0.001). On further analysis using the Contal and O’Quigley technique, a DNI cut-off of >5.3% at 12 h after ED admission was significantly associated with an increased risk of 30-day mortality (HR, 18.11; 95% CI, 6.99–46.94; *p* < 0.001) among patients admitted to an ICU with severe trauma. Compared to the derived Harrell C-index, the C-statistic of the DNI at 12 h was not statistically inferior to those of other markers (i.e., it was similar to the SOFA, APACHE II, ISS, lactate at 24 h, and PT at 12 h). Conversely, the C-statistic of the DNI at 12 h was statistically superior to those of lactate, PT, and tCO_2_ on ED admission (Fig. 4B and Supplement 3).

### The prognostic value of DNI when combined with conventional risk factors

The IDI and NRI are indicators of improvements following reclassification in a nested model, and can thus determine whether the predictive power of a combination of traditional factors is improved upon DNI inclusion. The addition of the DNI at 12 h after ED admission yielded a significantly positive IDI with respect to predicting 30-day mortality; the continuous NRI was also positive and was significant for the DNI at 12 h. Additionally, the diagnostic performance was significantly improved by adding DNI to the reference model for predicting MODS according to the IDI and NRI (Table 4).

**Table 4:** Comparison of the performance of the survival models (A) and multiple organ dysfunction syndrome (B) with and without the delta neutrophil index (DNI) by Harrell’s C-index, area under the curve operating characteristic (AUROC), integrated discrimination improvement (IDI), and continuous net reclassification improvement (NRI).

Prediction model	30-day mortality						
	Harrell’s C-index (95% CI)	Difference of C-index (95% CI)	*P*	IDI (95% CI)	*P*	NRI (95% CI)	*P*
**(A)**							
Reference(Ref.) model	0.917 (0.880–0.952)						
Ref. model + DNI Time-0	0.916 (0.875–0.956)	−0.001 (−0.016–0.011)	0.868	0.015 (−0.003–0.053)	0.098	0.117 (−0.217–0.267)	0.374
Ref. model + DNI Time-12	0.923 (0.885–0.958)	0.006 (−0.010–0.022)	0.453	0.063 (0.011–0.116)	0.012*	0.631 (0.244–0.721)	0.01*
Ref. model + DNI Time-24	0.895 (0.841–0.950)	−0.022 (−0.057–0.012)	0.196	0.028 (−0.010–0.110)	0.128	0.014 (−0.308–0.311)	0.907
Ref. model + DNI Time-48	0.892 (0.830–0.955)	−0.025 (−0.072–0.023)	0.317	0.112 (0.006–0.226)	0.040*	−0.022 (−0.269–0.517)	0.999
**Prediction model**	**Multiple organ dysfunction syndrome**						
	**AUROC (95% CI)**	**Difference of AUROC (95% CI)**	***P***	**IDI (95% CI)**	***P***	**NRI (95% CI)**	***P***
**(B)**							
Reference(Ref.) model	0.858						
Ref. model + DNI Time-0	0.859	0.001(−0.001–0.002)	0.475	0(−0.001–0.001)	0.886	0.004(−0.003–0.011)	0.317
Ref. model + DNI Time-12	0.889	0.031(−0.004–0.067)	0.086	0.048(0.012–0.085)	0.009*	0.207(0.108–0.307)	<0.001*
Ref. model + DNI Time-24	0.910	0.052(0.005–0.100)	0.031*	0.162(0.089–0.234)	<0.001*	0.262(0.133–0.390)	<0.001*
Ref. model + DNI Time-48	0.911	0.053(0.014–0.092)	0.008*	0.133(0.061–0.206)	<0.001*	0.283(0.142–0.344)	<0.001*

Harrell’s C-index showed discriminative abilities for the risk stratification of 30-day mortality (statistical information in Supplementary Table 2).

*P < 0.05.

Reference model = ISS + Acute Physiology and Chronic Health Evaluation (APACHE) II score + amount of transfused packed red blood cells + treatment modality + prothrombin time + arterial lactate level.

DNI, delta neutrophil index. *P < 0.05.

Reference model = ISS score + APACHE II score + amount of transfused packed red blood cells + treatment modality + prothrombin time + arterial lactate level + serum aspartate transaminase level.

DNI, delta neutrophil index. *P < 0.05.

### The effect of early significant intervention on the predictability of DNI values

Of the 366 patients, 213 (58.2%) underwent at least one of the early significant interventions (transfusion, n = 173, embolization, n = 66, and surgery, n = 101) within 12 hours after ED admission. We compared the predictability of DNI values for the development of MODS and 30-day mortality between patients who underwent at least one of the early significant interventions within 12 hours after ED admission and patients who received conservative management. There was no significant difference of the predictability of DNI values for the development of MODS between intervention (0.806 (95% CI: 0.734–0.879)) and conservative (0.846 (95% CI: 0.732–0.960)) groups (p = 0.563)). There was also no significant difference for predictability of DNI values for the 30-day mortality between the intervention (0.841 (95% CI: 0.784–0.884)) group and the conservative (0.917(95% CI: 0.883–0.98)) group (p = 0.157). Consequently, the predictability of DNI values in patients who underwent early significant intervention was similar to the total enrolled patient population and patients who received conservative management (Supplements 4 and 5).

## Discussion

We demonstrated that an increased DNI value, which reflects the fraction of immature granulocytes, was a significant independent predictor of the development of MODS and 30-day mortality. Despite advances in scoring systems and laboratory markers, the early recognition of severe complications such as the development of MODS and risk stratification for late death in traumatic injury remains challenging. The SOFA was originally developed as a descriptive score^23^; its final score is based on the degree of dysfunction of six organ systems as measured via several physiologic and laboratory results^23^. Although the SOFA is used to determine multiple organ failure (MOF) in trauma, it does not propose a cut-off value for MOF development^23^. The APACHE II score estimates mortality in the ICU based on a number of laboratory values and indicators of both acute and chronic diseases^24^. The ISS as an anatomical scoring system that provides an overall score for patients with multiple injuries and correlates linearly with mortality^24^. However, it is often difficult to fully determine the extent of injury before thorough investigation (and if required, surgery).

Neither the SOFA nor APACHE II scoring system predicts MODS after injury^24^. Durham *et al*. demonstrated that MOF was associated only with APACHE III; total blood products transfused in the first 24 h, and lactate levels at 24 h^25^. Our data showed that the AUCs of the DNI at 12 and 24 h were not statistically inferior to those of the APACHE II, total transfused packed red blood cell volumes, or lactate level at 24 h. Considering the efforts required to predict severity, including serial measurements over time, these scoring systems are excessively complicated for patients with trauma^26^.

As a screening tool for severely traumatized patients, we found that the DNI at 12 h after ED admission could predict the development of MODS and short-term mortality earlier than several other scoring systems. Inflammatory cytokines and C-reactive protein are also able to predict the development of organ failure with a high level of accuracy in traumatized patients^27^; even before clear clinical symptoms develop, interleukin-6 (IL-6) kinetics in the first days of hospitalization may predict the development of MOF^27^. However, it is difficult to estimate the development of MOF using levels of C-reactive protein (that is increased in response to (IL-6 secretion) 24 h after admission^27^. Although several pro-inflammatory cytokines like IL-6 can be usefully applied in clinical situations, additional costs, time, and new equipment must be considered for single or repeated measurements to predict the severity of the patients’ conditions. Recent studies have explored the DNI as an adjunct predictor because inflammatory markers that can be measured rapidly, easily, serially, and inexpensively are an unmet need^9,26^. The automatically calculated DNI is a more prompt and accurate method than manually counting immature granulocytes^10,14^. Several studies have shown that an increased DNI is associated with poor outcomes in specific disease conditions and can predict the severity of infection and sterile inflammation^9–11,14,15^. Although the pathophysiologic mechanisms by which DNI values are associated with severity of disease are not fully understood, several studies have elucidated the mechanisms by which immature granulocytes are released early and rapidly during severe inflammation^9,15,26^.

In the present study, we found that DNI values of >3.25% and >5.3% at 12 h post-admission were significant predictors of the development of MODS and 30-day mortality, respectively, in our trauma patients. Park *et al*. reported that severe sepsis and septic shock were significantly increased in patients with DNI > 6.5% in the first 24 h after ICU admission^11^. Yune *et al*. also demonstrated that a DNI > 10.5% on day 1 was associated with a higher 30-day mortality rate after surviving out-of-hospital cardiac arrest^14^. Taken together, the data show that the DNI value reflects the severity of diseases associated with systemic and sterile inflammation. The use of the DNI, which can be measured rapidly, easily, and inexpensively, can be used to assess the severity of several diseases because of the benefit of being automatically analysed as part of the complete blood count. Moreover, the present study revealed that the DNI value at 12 h post-ED admission predicted 30-day mortality with a reliability similar to those of the SOFA, APACHE II, ISS score, and lactate level at 24 h. Additionally, the DNI value 24 h post-admission was not inferior to the other factors (except for the SOFA) in predicting 30-day mortality. The DNI reflects the severity that is associated with MODS and short-term mortality, and produces predictive values that are statistically similar to several other scoring systems. Therefore, we cautiously propose that DNI values can be an ancillary test for the early prediction of injury/disease severity 12 h before other scoring systems such as the ISS, SOFA, and APACHE II, and is a serially measurable marker for monitoring severity over time in patients with trauma.

This study had several limitations. First, despite using the prospective TRAUMA CP registry that had a standardized and predetermined protocol at our institution, our data from a single, tertiary, academic hospital were analysed retrospectively. We could not mandatorily and serially measure certain classification systems such as the Trauma and Injury Severity Score (TRISS), New Injury Severity Score, and APACHE III, nor could we investigate other inflammatory mediators such as tissue necrosis factor and other pro-inflammatory cytokines that are known indicators of trauma severity in our TRAUMA CP protocol^28^. Therefore, it was difficult to control for confounding factors, which increased the possibility of selection bias. However, we did investigate the TRISS (ISS plus the revised trauma score including systolic blood pressure, respiratory rate, and Glasgow Coma Scale) at limited time points. We also constructed a multivariable regression model considering the risk of multicollinearity and the possibility of overestimation or underestimation. We used the ISS, SOFA, and APACHE II scores as factors in this model because these scoring systems inherently introduced variability by not being applied consistently in our trauma patients. Sudarsanam *et al*. demonstrated that the APACHE II and APACHE III scores were strongly associated, with a correlation score of 0.8^29^. In future studies, to validate the prognostic potential of DNI, a prospective study is needed to compare the predictive power of DNI values and other reliable indicators of organ dysfunction (such as APACHEIII, New Injury Severity Score, and cytokines) as an independent risk factor to add to reference prediction models. A second limitation was that we excluded patients admitted to the NCU to achieve homogeneity; hence, patients with severe brain injury may not have been included in this study. However, severe injury isolated to the brain is closely associated with higher mortality. Third, in an evaluation of the usefulness of a new marker when the marker is added to reference models with sufficient predictive power or standard risk factors, the application of AUC may be too conservative and the NRI and IDI may be overestimated. The statistical interpretation needs to be carefully considered due to the limitations of the two statistical methods.

Finally, our study did not clarify the precise mechanisms by which the DNI is associated with severity after traumatic injury. Large, prospective, randomized clinical trials, as well as additional targeted investigations, are required to address each of these limitations.

In conclusion, an increased DNI value is an independent predictor of MODS development and 30-day mortality in trauma patients admitted to an ICU. The DNI is routinely and easily measurable as part of the complete blood count without additional cost or time, and can therefore be considered a suitable parameter for the early estimation of the severity of a traumatic injury.

## Methods

### Study population and the TRAUMA critical pathway (CP)

A retrospective cohort single-centre study was performed in the emergency department (ED) of an urban, university-affiliated, tertiary referral medical centre with an annual census of approximately 85,000 visits. The study was approved by the institutional review board (No. 3-2017-0188) of Yonsei University Health System, which waived the requirement for written informed consent because of the retrospective nature of the study. All methods were performed in accordance with the relevant guidelines and regulations. This study enrolled eligible adult patients who were admitted to the ICU after enrolment in the TRAUMA CP of the ED between January 1, 2011, and May 31, 2017. Figure 1 shows the enrolment, TRAUMA CP alerting criteria, and clinical outcome data of patients suspected of having sustained severe trauma. To validate the usefulness of serial DNI measurements over time, we excluded patients who transferred from another hospital before admission to our institution’s ED, those who transferred out to another hospital within the first 96 h, those discharged against medical advice, and those who died in the ED. Additional exclusion criteria were a history of haematological malignancy or autoimmune disease, admission to the independent neurosurgical care unit (NCU) for isolated head injuries, and current pregnancy; moreover, those who died within the first 96 h after injury were not included in MODS-related analyses based on predetermined guidelines^1,23^. Considering the differences in intensive management, we excluded trauma patients treated in the NCU to maintain the homogeneity of the study cohort.

Our institution schedules specialized traumatologists and interventional radiologists to be on call 24 h/day, 7 days/week, to treat patients who arrive at the ED requiring emergency surgical and radiological interventions. In 2011, our TRAUMA CP was designed and implemented for the effective management of adult patients (≥18 years) suspected of having sustained severe trauma to reduce unnecessary in-hospital time delays through a computerized provider order entry-based alert system, short message service, and simple standing orders through the activation stage^28^. TRAUMA CP records were prospectively obtained based on a predetermined protocol. In the triage area of the ED, physicians, nurses, or emergency medical technicians screened candidates for the TRAUMA CP program as soon as feasible using the Advanced Trauma Life Support (ATLS) guidelines^28^. When a patient had at least one of seven warning mechanisms and one of 10 clinical suspicions of severe trauma (known as the TRAUMA CP alerting criteria) on ED admission, an ED physician in the triage area activated the TRAUMA program by selecting the activation icon on the order-entry window (Fig. 1). The computerized provider order entry system highlights the name of the patient in purple and simultaneously submits medical orders according to a predetermined program. According to the predetermined protocol based on the ATLS guidelines, the traumatologists and emergency physicians immediately evaluate the patient and simultaneously administer treatment^28^. Even if the system was activated, we excluded patients with minor injuries from our program to preserve the cost-effective use of medical resources and limit radiation hazards. In the emergency setting, medical staff reassessed each patient by performing a physical examination and ‘focused assessment with sonography for trauma’, and decided whether to include the patient in this program^28^. This protocol was applied for 24 h/7 days, whereupon severe trauma patients received standard management from the ED to the ICU.

#### Data collection

We collected baseline characteristics including sex, age, previous medical history, mechanism of injury, mental status on arrival, vital signs, laboratory values, Injury Severity Score (ISS), and follow-up data for all patients based on a predetermined protocol. We also investigated whether packed red blood cells, fresh frozen plasma, and platelets were transfused in the first 24 h, and noted the main types of emergency treatment performed within 24 h of ED admission (including emergency surgery, angioembolization, and conservative care). The Sequential Organ Failure Assessment (SOFA) and Acute Physiology and Chronic Health Evaluation (APACHE) II were obtained from the TRAUMA CP records to determine the clinical severity of each patient’s condition. Additional data were extracted from electronic sources on radiology, ICU progress notes, nursing charts, and discharge summaries.

The DNI values were obtained for all patients within 10 minutes of ED admission as well as 12 ± 3 h, 24 ± 3 h, and 48 ± 6 h after admission. The DNI for each patient was determined using venous blood in ethylenediaminetetraacetic acid (EDTA)-containing vacutainers on presentation to the ED. To assess the DNI, we used the same type of haematology analyser (ADVIA 2120; Siemens, Forchheim, Germany) used for the analysis of the complete blood count. We also conducted other laboratory tests including measuring white blood cell counts and levels of blood urea nitrogen, creatinine, electrolytes, alanine transaminase, and lactate at the time of ED admission.

#### DNI measurement

The analysers used to determine DNI were optical systems based on a cytochemical myeloperoxidase tungsten-halogen channel (which measures and differentiates neutrophils, eosinophils, lymphocytes, monocytes, and large unstained cells based on size and myeloperoxidase staining intensity), and a laser-diode channel (that calculates, classifies, and counts cell types based on lobularity/nuclear density and size). The DNI was then calculated by subtracting the fraction of mature polymorphonuclear neutrophils from the sum of the myeloperoxidase-reactive cells, thus deriving the circulating immature granulocytes as the leukocyte sub-fraction^10,12,14^.

#### Clinical endpoints

Our primary endpoint was the development of MODS during in-hospital admission, which was defined as a Sequential Organ Failure Assessment Score of 6 or more on two or more consecutive days at least 48 h after ED admission^5^. Additionally, the secondary endpoint was all-cause mortality within 30 days of ED admission after sustaining trauma.

### Statistical analysis

Demographic and clinical data are presented as percentages or frequencies, medians (interquartile ranges), and means ± standard deviations, as appropriate. We compared categorical variables using the χ^2^ test or Fisher’s exact test and continuous variables using a two-sample *t*-test or the Mann–Whitney U-test. Using a linear mixed model and repeated measures covariance pattern with unstructured covariance within patients, we evaluated significant differences between groups over time. Two fixed effects represented the clinical effect (level: survival vs. death by day 30 and development vs. non-development of MODS) and time effect (time: DNI determined on admission and at 12 h, 24 h, and 48 h after ED admission). Differences in clinical effect over time were analysed by the formula “clinical effect × time”.

We also measured significant differences concerning the development of MODS. Univariate Cox proportional hazards regression and logistic regression analyses were used to evaluate relationships among demographic characteristics and clinical data for all-cause mortality at 30 days and the development of MODS. Multivariable Cox proportional hazards regression that integrated the major covariates (variables with a *p*-value < 0.05) identified by our univariate Cox proportional hazards regression analysis was used to determine potential independent predictors of 30-day mortality by considering time-to-event data. Additionally, multivariable logistic regression analysis was performed to assess potential independent predictors of the development of MODS. The results of multivariable Cox proportional hazards regression analyses are expressed as hazard ratios (HRs) and 95% confidence intervals (CIs). Those of multivariable logistic regression analysis are represented as odds ratios (ORs) and 95% CIs. We determined the area under the curve (AUC) to assess the ability of the DNI to predict the development of MODS using receiver operating characteristic (ROC) curves. We assessed the optimal cut-off for the DNI using Youden’s method for discriminating the development of MODS in trauma patients admitted to an ICU. Next, ROC curves were constructed to assess the diagnostic performance of the DNI and other clinical parameters, and the AUCs were calculated and compared. We also performed net reclassification improvement (NRI) and integrated discrimination improvement (IDI) to analyse the degree to which the addition of DNI to the reference model improved its predictive ability^30^.

We constructed Kaplan-Meier survival curves using 30-day mortality data, and compared groups using the log-rank test. The optimal cut-off values for the dichotomization of the clinical outcome variable were estimated based on time-to-event data using the technique devised by Contal and O’Quigley^14,31^. The optimal cut-off point was selected by maximizing the HR. To investigate the additional predictive power of the DNI at each time point, Harrell’s C-index was assessed to examine the time-dependent discriminatory ability against SOFA, APACHE II, and ISS on ICU admission; lactate, total CO_2_ and prothrombin time on ED admission; and lactate and prothrombin time 24 h after ED admission versus the ability of DNI to predict 30-day mortality^32^. To assess the 95% CI and *p*-values for the IDI and continuous NRI, as well as the C-index and differences between models, a standard bootstrap method was used with resampling performed 1,000 times. We also assessed the continuous NRI and IDI at the final follow-up visit (30 days) to assess the improvement in performance of the survival model when DNI (as an additional parameter) is included^9,33,34^. To identify the effect of early significant intervention including transfusion, embolization, and surgery on the predictability of DNI values, we reviewed medical records to identify patients who received significant intervention before obtaining DNI within 12 h after ED admission. We compared the predictability of DNI values for the development of MODS and the 30-day mortality between those that received early intervention and those that had more conservative management using ROC curves with Delong method and Harrell’s C-index. Statistical analyses were performed using SAS, version 9.2 (SAS Institute Inc., Cary, NC); R software, version 3.2.5 for Windows (the R foundation for statistical computing, Vienna, Austria; http://www.R-project.org/); and MedCalc, version 12.7.0 (MedCalc Software, Ostend, Belgium). A *p*-value < 0.05 was considered significant.

### Ethics approval and consent to participate

The study was approved by the institutional review board (No. 3-2017-0188) of Yonsei University Health System, which waived the requirement for written informed consent because of the retrospective nature of the study. All methods were performed in accordance with the relevant guidelines and regulations.

## Electronic supplementary material

Supplementary Information

## Data Availability

The datasets generated during and/or analysed during the current study are available from the corresponding author on reasonable request.
