# Supplementary material for: The delta neutrophil index predicts development of multiple organ dysfunction syndrome and 30-day mortality in trauma patients admitted to an intensive care unit: a retrospective analysis
Source: Sci Rep. 2018 Nov 30;8:17515. doi: 10.1038/s41598-018-35796-4 (PMC6269472; doi:10.1038/s41598-018-35796-4)

Supplementary Information

The delta neutrophil index predicts development of multiple organ dysfunction syndrome and 30-day mortality in trauma patients admitted to an intensive care unit: a retrospective analysis

Taeyoung Kong, MD[1,2]; Yoo Seok Park, MD, PhD[1]; Hye Sun Lee, PhD[3]; Sinae Kim, MS[3]; Jong Wook Lee, MD[4]; Je Sung You, MD, PhD[1]; Hyun Soo Chung, MD, PhD[1]; Incheol Park, MD, PhD[1]; Sung Phil Chung, MD, PhD[1]*

1. Department of Emergency Medicine, Yonsei University College of Medicine, Seoul, Republic of Korea

2. Department of Emergency Medicine, Graduate School of Medicine, Kangwon National University, Chuncheon, Republic of Korea

3. Department of Research Affairs, Biostatistics Collaboration Unit, Yonsei University College of Medicine, Seoul, Republic of Korea

4. Department of Laboratory Medicine, Konyang University Hospital, Daejeon, Republic of Korea

Corresponding author: Je Sung You, MD, PhD*

Department of Emergency Medicine, Yonsei University College of Medicine

211 Eonju-Ro, Gangnam-Gu, Seoul 135-720, Republic of Korea

E-mail: youjsmd@yuhs.ac /TEL: 82-2-2019-3030, FAX: 82-2-2019-4820

Supplement 1. Univariate Cox proportional hazards regression analysis for predictors of 30-day mortality and univariate logistic regression analysis for predictors of development of multiple organ dysfunction syndrome**.**

Variables	30-day mortality		Development of MODS	
	HR (95% CI)	P	OR (95% CI)	P
Age (per 1years)	1.024 (1.005-1.042)	0.011*	1.004 (0.990-1.019)	0.569
Male (vs Female)	0.912 (0.451-1.846)	0.798	1.488 (0.766-2.893)	0.241
BMI (per 1kg/m^2)	1.034 (0.950-1.124)	0.44	1.025 (0.950-1.105)	0.526
ISS (per 1point)	1.069 (1.049-1.089)	<0.001*	1.085 (1.056-1.116)	<0.001*
APACHE II score (per 1point)	1.152 (1.116-1.190)	<0.001*	1.152 (1.103-1.204)	<0.001*
SOFA score (per 1point)	1.474 (1.351-1.608)	<0.001*	1.732 (1.527-1.965)	<0.001*
**Initial vital sign**				
Mean blood pressure (per 1mmHg)	0.964 (0.954-0.974)	<0.001*	0.978 (0.966-0.990)	<0.001*
Heart rate (per 1bpm)	0.986 (0.974-0.998)	0.022*	1.007 (0.995-1.020)	0.236
Respiratory rate (per 1bpm)	0.922 (0.864-0.984)	0.014*	0.946 (0.885-1.012)	0.105
Body temperature (per 1°C)	0.600 (0.386-0.931)	0.023*	0.392 (0.253-0.607)	<0.001*
Mental change (vs Alert)	6.003 (3.142-11.468)	<0.001*	5.922 (3.140-11.166)	<0.001*
**Mechanism [n (%)]**				
Motor vehicle accident at high speed	Reference (1)		Reference (1)	
Ejection or rollover of vehicle	1.081 (0.112-10.396)	0.946	1.020 (0.234-4.438)	0.979
Pedestrian stuck by moving vehicle	2.398 (0.703-8.184)	0.163	0.737 (0.309-1.760)	0.492
Bicycle accident > 20km impact	4.571 (0.764-27.367)	0.096	0.567 (0.061-5.277)	0.618
Fall > 3m	1.815 (0.500-6.596)	0.365	0.785 (0.319-1.932)	0.598
Motorcycle accident	0.486 (0.081-2.908)	0.429	0.449 (0.156-1.293)	0.138
Crushing injury	0.771 (0.080-7.413)	0.822	0.907 (0.245-3.357)	0.883
Stab wound	0.612 (0.064-5.882)	0.671	0.309 (0.062-1.547)	0.153
**Comorbidity**				
Hypertension	1.297 (0.660-2.547)	0.45	1.262 (0.684-2.329)	0.456
Diabetes mellitus	1.517 (0.716-3.215)	0.277	1.140 (0.537-2.418)	0.734
Chronic pulmonary disease	0.840 (0.202-3.493)	0.811	1.139 (0.369-3.513)	0.821
Cardiovascular disease	1.277 (0.392-4.157)	0.685	1.364 (0.486-3.829)	0.556
Old cerebrovascular accident	0.653 (0.039-11.035)	0.768	0.797 (0.094-6.741)	0.835
Malignancy	1.119 (0.153-8.162)	0.912	0.272 (0.013-5.692)	0.402

Chronic liver disease	4.756 (1.143-19.788)	0.032*	4.931 (0.681-35.722)	0.114
Chronic kidney disease	0.653 (0.039-11.035)	0.768	0.797 (0.094-6.741)	0.835
**Treatment within 24 hours**				
Surgery	3.003 (1.545-5.837)	0.001*	2.130 (1.213-3.743)	0.009*
Embolization	2.087 (1.048-4.153)	0.036	2.027 (1.066-3.855)	0.031*
Conservative management	0.316 (0.149-0.670)	0.003*	0.456 (0.255-0.813)	0.008*
**Transfusion in first 24 hours**				
Packed red blood cells (per 1U)	1.068 (1.051-1.085)	<0.001*	1.176 (1.115-1.241)	<0.001*
Fresh Frozen Plasma (per 1U)	1.090 (1.067-1.113)	<0.001*	1.205 (1.131-1.283)	<0.001*
Platelet concentrate (per 1U)	1.087 (1.063-1.112)	<0.001*	1.208 (1.136-1.285)	<0.001*
**Laboratory data**				
White blood cell count (per 10^3/μL)	0.944 (0.877-1.016)	0.122	0.963 (0.908-1.021)	0.206
Hemoglobin (per 1g/dL)	0.845 (0.751-0.949)	0.005*	0.948 (0.845-1.065)	0.368
Platelet count (per 10^3/μL)	0.996 (0.992-1.000)	0.074	0.997 (0.993-1.000)	0.071
PT (per 1 INR)	4.214 (2.341-7.587)	<0.001*	5.598 (1.564-20.033)	0.008*
BUN (per 1mg/dL)	1.029 (0.993-1.067)	0.118	0.990 (0.947-1.035)	0.657
Creatinine (per mg/dL)	1.243 (1.006-1.534)	0.044*	1.338 (0.964-1.857)	0.082
AST (per 1 IU/L)	1.001 (1.000-1.003)	0.056	1.002 (1.000-1.003)	0.014*
ALT (per 1 IU/L)	1.001 (1.000-1.003)	0.104	1.002 (1.000-1.004)	0.032*
Lactate (per 1mmol/L)	1.234 (1.159-1.314)	<0.001*	1.318 (1.196-1.452)	<0.001*
Potassium (per 1mmol/L)	1.346 (0.780-2.321)	0.286	0.883 (0.531-1.468)	0.63
tCO2 (per 1mmol/L)	0.851 (0.788-0.918)	<0.001*	0.841 (0.776-0.911)	<0.001*
DNI Time-0 (per 1%)	0.889 (0.753-1.051)	0.168	0.999 (0.937-1.065)	0.973
DNI Time-12 (per 1%)	1.049 (1.031-1.068)	<0.001*	1.094 (1.051-1.139)	<0.001*
DNI Time-24 (per 1%)	1.055 (1.034-1.076)	<0.001*	1.199 (1.122-1.281)	<0.001*
DNI Time-48 (per 1%)	1.060 (1.039-1.081)	<0.001*	1.185 (1.098-1.278)	<0.001*

*P<0.05

BMI, body mass index; ISS, injury severity score; APACHE II, Acute Physiology and Chronic Health Evaluation; SOFA, sequential organ failure assessment; PT, prothrombin time; INR, international normalized ratio; BUN, blood urea nitrogen; AST, aspartate aminotransferase; ALT, alanine aminotransferase; DNI, delta neutrophil index.

Supplement 2. Comparing scoring systems and biomarkers for predicting development of multiple organ dysfunction syndrome using the area under the curve**.**

Variables	AUC (95% CI)	*P*	*P* (vs.DNI Time12)	*P* (vs.DNI Time24)	*P* (vs.SOFA Time24)	*P* (vs.APACHEII)	*P* (vs.ISS)	*P* (vs.Lactate Time 0)	*P* (vs.Lactate Time24)	*P* (vs.Amount of pRBC)
DNI Time 12	0.809 (0.743-0.875)	<0.001*	Ref.	0.516	0.006*	0.197	0.143	0.088	0.33	0.382
DNI Time 24	0.836 (0.764-0.908)	<0.001*	0.516	Ref.	0.056	0.057	0.037*	0.026*	0.849	0.121
SOFA score Time 24	0.911 (0.873-0.949)	<0.001*	0.006*	0.056	Ref.	<0.001*	<0.001*	<0.001*	0.029*	<0.001*
APACHE II score	0.739 (0.661-0.817)	<0.001*	0.197	0.057	<0.001*	Ref.	0.897	0.737	0.043	0.587
ISS	0.732 (0.654-0.810)	<0.001*	0.143	0.037	<0.001*	0.897	Ref.	0.889	0.018	0.49
Lactate Time 0	0.725 (0.645-0.804)	<0.001*	0.088	0.026*	<0.001*	0.737	0.889	Ref.	0.002*	0.336
Lactate Time 24	0.843 (0.782-0.904)	<0.001*	0.33	0.849	0.029	0.043*	0.018*	0.002*	Ref.	0.026*
Amount of pRBC	0.769 (0.692-0.846)	<0.001*	0.382	0.121	<0.001*	0.587	0.49	0.336	0.026*	Ref.

*P<0.05

AUC, area under the curve; CI, confidence interval; DNI, delta neutrophil index; SOFA, sequential organ failure assessment; APACHE II, Acute Physiology and Chronic Health Evaluation; ISS, injury severity score; pRBC, packed red blood cells.

Supplement 3. Comparison of scoring systems and biomarkers for the prediction of 30-day mortality using Harrell's C-index.

Variables	Harrell's c index (95% CI)	*P*	*P* (vs. DNI Time 12)	*P* (vs.DNI Time 24)	*P* (vs.SOFA Time24)	*P* (vs.APACHE)	*P* (vs. ISS)	*P* (vs.Lactate Time 0)	*P* (vs. Lactate Time 24)	*P* (vs. PT Time 0)	*P* (vs. tCO2 Time 0)	*P* (vs. Amount of pRBC)
DNI Time 12	0.877 (0.834-0.917)	<0.001*	Ref.	0.084	0.134	0.729	0.052	0.007*	0.442	<0.001*	<0.001*	0.235
DNI Time 24	0.776 (0.635-0.884)	<0.001*	0.084	Ref.	0.029*	0.127	0.599	0.953	0.069	0.114	0.149	0.34
SOFA score Time 24	0.909 (0.868-0.948)	<0.001*	0.134	0.029*	Ref.	0.027*	0.005*	<0.001*	0.603	<0.001*	<0.001*	0.022*
APACHE score	0.868 (0.817-0.913)	<0.001*	0.729	0.127	0.027*	Ref.	0.127	0.002*	0.35	<0.001*	<0.001*	0.469
ISS score	0.807 (0.74-0.868)	<0.001*	0.052	0.599	0.005*	0.127	Ref.	0.475	0.041*	0.009*	0.012*	0.457
Lactate Time 0	0.772 (0.695-0.845)	<0.001*	0.007*	0.953	<0.001*	0.002*	0.475	Ref.	<0.001*	0.064	0.015*	0.18
Lactate Time 24	0.897 (0.836-0.944)	<0.001*	0.442	0.067	0.603	0.35	0.041*	<0.001*	Ref.	<0.001*	<0.001*	0.088
PT Time 0	0.659 (0.561-0.757)	0.002*	<0.001*	0.114	<0.001*	<0.001*	0.009*	0.06	<0.001*	Ref.	0.839	0.004*
tCO2 Time 0	0.672 (0.58-0.755)	<0.001*	<0.001*	0.149	<0.001*	<0.001*	0.012*	0.015*	<0.001*	0.839	Ref.	0.004*
Amount of pRBC	0.839 (0.769-0.902)	<0.001*	0.235	0.34	0.022*	0.469	0.457	0.18	0.088	0.004*	0.004*	Ref.

*P<0.05

CI, confidence interval; DNI, delta neutrophil index; SOFA, sequential organ failure assessment; APACHE II, Acute Physiology and Chronic Health Evaluation; ISS, injury severity score; PT, prothrombin time; pRBC, packed red blood cells..

Supplement 4. Multivariable Cox proportional hazards regression analysis for predictors of 30-day mortality (A) and multivariable logistic regression analysis for predictors of development of multiple organ dysfunction syndrome (B) according to performed intervention in first 12 hours.

(A)

Variable	Surgery within 12H		Embolization within 12H		Transfusion within 12H		Any intervention within 12H	
	HR (95% CI)	*P*	HR (95% CI)	*P*	HR (95% CI)	*P*	HR (95% CI)	*P*
ISS (per 1point)	1.035(0.986-1.086)	0.166	1.033(0.981-1.087)	0.22	1.041(1.012-1.070)	0.005*	1.039(1.010-1.068)	0.007*
APACHE II score (per 1point)	1.067(0.962-1.183)	0.221	1.087(0.927-1.275)	0.305	1.083(1.018-1.153)	0.012*	1.090(1.026-1.158)	0.005*
Transfusion of pRBC (per 1unit)	1.019(0.984-1.054)	0.288	1.035(0.973-1.101)	0.274	1.028(1.002-1.054)	0.033*	1.028(1.003-1.054)	0.03*
Prothrombin time (per 1 INR)	2.092(0.606-7.219)	0.243	1.009(0.116-8.806)	0.993	1.012(0.362-2.831)	0.982	1.002(0.365-2.747)	0.997
Lactate (per 1mmol/L)	1.098(0.903-1.335)	0.35	0.976(0.712-1.337)	0.878	1.055(0.936-1.190)	0.379	1.056(0.939-1.187)	0.367
DNI Time 12 (per 1 %)	1.037(1.000-1.075)	0.048*	1.080(1.032-1.130)	0.001*	1.055(1.027-1.083)	<0.001*	1.053(1.026-1.081)	<0.001*

*P<0.05

ISS, injury severity score; APACHE II, Acute Physiology and Chronic Health Evaluation; pRBC, packed red blood cells; INR, international normalized ratio; DNI, delta neutrophil index.

(B)

Variable	Surgery within 12H		Embolization within 12H		Transfusion within 12H		Any intervention within 12H	
	OR (95% CI)	*P*	OR (95% CI)	*P*	OR (95% CI)	*P*	OR (95% CI)	*P*
ISS (per 1point)	1.046(0.985-1.110)	0.14	0.992(0.872-1.129)	0.907	1.023(0.979-1.069)	0.315	1.036(0.993-1.082)	0.102
APACHE II score (per 1point)	1.011(0.865-1.182)	0.889	1.157(0.912-1.468)	0.229	1.015(0.943-1.093)	0.686	1.024(0.952-1.102)	0.52
Transfusion of pRBC (per 1unit)	1.165(1.029-1.318)	0.016*	1.228(1.029-1.465)	0.023*	1.124(1.046-1.207)	0.002*	1.133(1.054-1.217)	<0.001*
AST (per 1 IU/L)	1.004(1.000-1.009)	0.078	1.002(0.999-1.006)	0.222	1.003(1.000-1.006)	0.056	1.002(1.000-1.005)	0.084
Prothrombin time (per 1 INR)	0.208(0.004-12.213)	0.45	32.291(0.343-3039.573)	0.134	1.343(0.308-5.847)	0.695	1.313(0.301-5.721)	0.717
Lactate (per 1mmol/L)	1.246(0.876-1.772)	0.221	0.948(0.663-1.357)	0.772	1.112(0.955-1.296)	0.172	1.115(0.959-1.296)	0.157
DNI Time-12 (per 1 %)	1.063(1.017-1.112)	0.008*	1.133(1.015-1.263)	0.026*	1.070(1.026-1.117)	0.002*	1.064(1.026-1.104)	<0.001*

*P<0.05

ISS, injury severity score; APACHE II, Acute Physiology and Chronic Health Evaluation; pRBC, packed red blood cells; AST, aspartate aminotransferase; INR, international normalized ratio; DNI, delta neutrophil index.

Supplement 5. Comparison of Harrell's C-index for the delta neutrophil index (DNI) for the prediction of 30-day mortality according to intervention within 12 hours (A); and comparison of the area under the curve (AUC) for the DNI when predicting the development of multiple organ dysfunction syndrome (MODS) according to intervention within 12 hours (B). The predictability of DNI values in patients who underwent early significant intervention was similar to the patients who received conservative management.

(A)

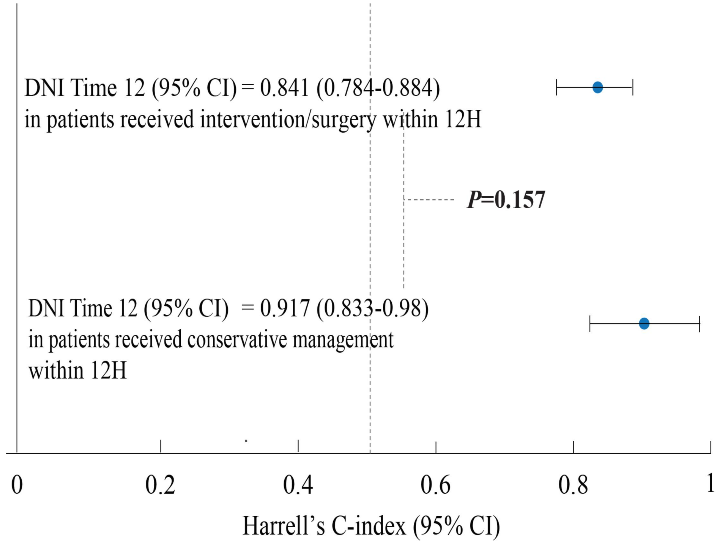

(B)

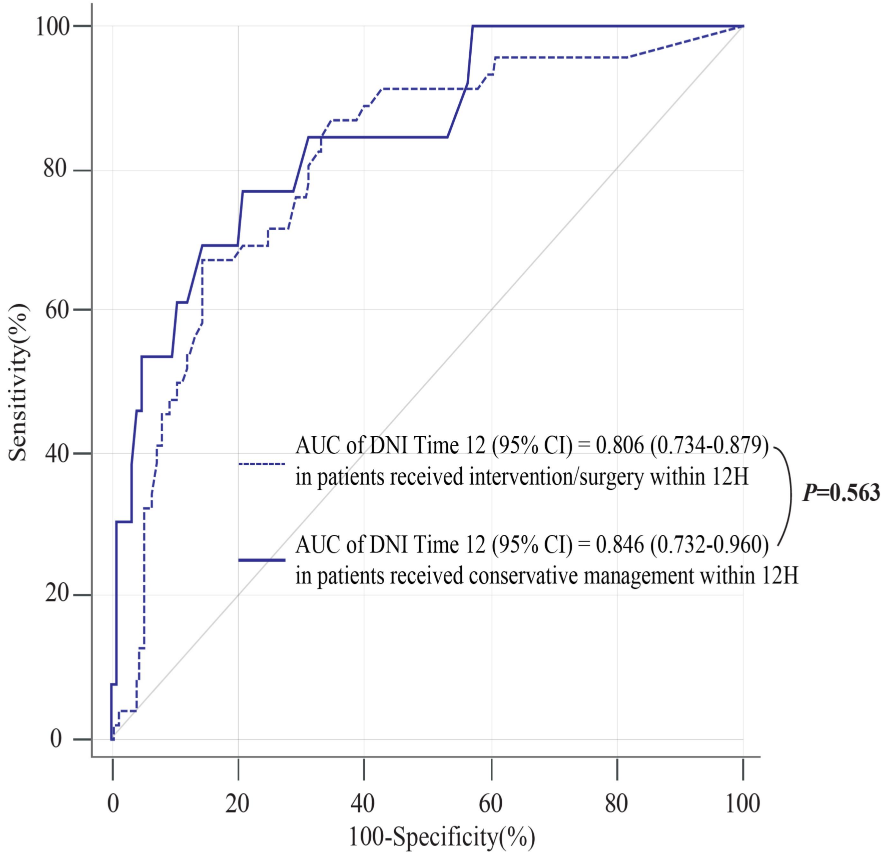

Supplement: Supplementary file 1 — Supplementary Information [file 41598_2018_35796_MOESM1_ESM.pdf]
